# Sleep patterns, plasma metabolites, and risk of incident osteoarthritis: a prospective cohort study

**DOI:** 10.1038/s41598-025-07711-1

**Published:** 2025-08-11

**Authors:** Hao Xia, Chen Meng, Rong Chen, Chao Mao, Chuan Li, Yongqing Xu

**Affiliations:** 1 Department of Orthopaedic, 920Th Hospital of Joint Logistics Support Force of Chinese People’s Liberation Army, 212 Daguan Road, Xishan District, Kunming, Yunnan China; 2 Institute of Translational Medicine, Medical College, Yangzhou University, Yangzhou, Jiangsu China; 3 Department of Rehabilitation Medicine, The First Affiliated Hospital, Sun Yat-Sen University, Guangzhou, Guangdong China; 4 Faculty of Applied Sciences, Macao Polytechnic University, Macao, SAR 999078 China; 5 Kunming Institute of Zoology, Chinese Academy of Sciences, No.17 Longxin Road, Kunming, Yunnan China

**Keywords:** Sleep Patterns, Osteoarthritis, Plasma metabolites, Cohort study, UK Biobank, Immunology, Biomarkers, Medical research, Molecular medicine, Risk factors

## Abstract

Although previous studies have assessed the effect of sleep traits on osteoarthritis (OA) risk, the association between the complex interplay of multiple sleep patterns and OA risk remains uncertain. We included participants who were free of OA at baseline based the UK Biobank. We evaluated the associations of five sleep behaviors with the risk of OA using Cox proportional hazard regression models. To explore the metabolic profile of sleep patterns, we regressed the sleep score on 167 standardized metabolites using ten iterations of LASSO model with ten-fold cross-validation. Restricted cubic splines (RCS) with four knots were used in the fully adjusted model to explore the potential non-linear association of sleep score and metabolic profile with OA risk. We discovered that individuals with poor sleep patterns experienced a notably higher incidence of OA (HR, 1.23, 95% CI, 1.18 to 1.28, *P* = 2.69 × 10^–23^). Furthermore, the risk of hand OA specifically was 1.29 times higher among those with poor sleep patterns compared to those with healthy sleep patterns (HR, 1.29, 95% CI, 1.12 to 1.49, *P* = 4.23 × 10^–4^). Individuals belonging to the highest quintile of metabolic score exhibited a 1.14-fold elevated risk of OA compared to those in the lowest quintile (HR, 1.14; 95% CI, 1.08 to 1.20; *P* = 3.09 × 10^–6^). Our findings have important public health implications as we provide an objective and more comprehensive evaluation of sleep patterns, and novel insights into the mechanisms linking sleep patterns and OA through metabolic profile.

## Introduction

Osteoarthritis (OA) is the most common chronic degenerative joint disorder, presenting with symptoms such as joint pain, deformities, and limited range of motion^1^. With the global population aging and obesity rates rising, the prevalence of OA is increasing steadily^2^. It is currently estimated that over 250 million people worldwide are affected by OA^2^. Moreover, studies on the economic burden suggest that the global per capita cost of managing OA exceeds $10,000, imposing a considerable strain on healthcare systems^3,4^. As a result, OA is expected to become an even more significant public health challenge in the coming decades.

Although the precise etiology and mechanisms of osteoarthritis (OA) remain unclear, evidence suggests that several risk factors—including obesity, aging, trauma, metabolic disorders, and sleep patterns—contribute to the onset and progression of OA^5–7^. Notably, recent studies have highlighted that sleep traits, such as insomnia, excessive daytime sleepiness, and abnormal sleep duration, are associated with an increased risk of OA^8–10^. For instance, a nationwide cross-sectional study of 11,540 participants identified a U-shaped relationship between sleep duration and OA, demonstrating a significant association between the two^11^. Additionally, a large case–control study involving 351,932 adults in the UK found a significant association between sleep disorders and OA (odds ratio [OR] = 1.25, 95% confidence interval [CI] = 1.22–1.29), consistent across all sex and age subgroups^9^. However, most previous studies have been cross-sectional, often focusing on the impact of individual sleep behaviors on OA risk without accounting for the complex interplay of multiple sleep patterns or conducting prospective analyses.

Growing evidence suggests that circulating serum metabolites play a significant role in the onset and progression of various diseases^12–14^. Metabolites—small molecules that function as intermediates or end products of cellular metabolism—reflect an organism’s metabolic state and may serve as valuable diagnostic biomarkers or therapeutic targets for disease^15^. Notably, several circulating metabolites have been linked to sleep behaviors and are considered potential mediators of the impact of sleep on health outcomes^16–18^. For instance, a nested case–control study reported consistently elevated lipid levels in women with poorer sleep quality^19^. Similarly, a study involving 277 participants identified widespread associations between sleep timing and metabolites related to lipid metabolism, including bile acids, carnitines, and fatty acids^20^. However, few studies have prospectively examined the causal relationship between metabolomic alterations and the risk of developing OA.

To address current research gaps, this study investigates the associations between nuclear magnetic resonance (NMR)-based circulating metabolic biomarkers and the risk of OA in participants from the UK Biobank (UKB). In addition, we developed a composite metabolic risk score to comprehensively evaluate the influence of metabolomic profiles on OA risk. Finally, for the first time, we identified the metabolomic signature associated with a healthy overall sleep pattern and prospectively analyzed its relationship with OA risk.

## Methods

### Study design and population

The UKB is a large-scale prospective cohort study that recruited approximately 500,000 participants between 2006 and 2010, aged 37 to 73 years at enrollment^21^. Participants attended one of 22 assessment centers across the United Kingdom, where they provided informed electronic consent and completed self-administered touchscreen questionnaires and computer-assisted interviews to collect comprehensive sociodemographic, lifestyle, and personal health information. In addition, they underwent a series of physical and functional assessments and provided biological samples, including blood, urine, and saliva^21^. The detailed descriptions of the study design and data collection procedures are available online (https://www.ukbiobank.ac.uk). The study granted access to the UK Biobank database, which received ethical approval from the North West Multi-Centre Research Ethics Committee (21/NW/0157). The present analyses were conducted under UK Biobank application number 193639.

### Ascertainment of sleep risk score

There were five sleep traits reported by participants including daytime sleepiness, insomnia, sleep duration, chronotype, and snoring in the UKB^22,23^. Subjective daytime sleepiness was assessed using the question, “How likely are you to doze off or fall asleep during the daytime when you don’t mean to”. For analysis, a binary variable for daytime sleepiness was created, categorizing responses as “never/rarely or sometimes” versus “often or all of the time.” Insomnia symptoms were evaluated based on the question, “Do you have trouble falling asleep at night or do you wake up in the middle of the night”. A binary insomnia variable was generated, classifying responses as “never/rarely” versus “sometimes/usually”. Sleep duration was recorded based on the question, “How many hours of sleep do you get in every 24 h”. Consistent with previous studies, sleep duration was categorized as normal (7–8 h/day), short sleep (< 7 h/day), and long sleep (≥ 9 h/day). Chronotype preference was determined by the question, “Do you consider yourself to be” with four response options: “definitely a morning person”, “more of a morning than an evening person”, “more of an evening than a morning person”, or “definitely an evening person”. For analysis, participants were further classified into a two group, “early chronotype” (including “morning person” and “more of a morning than an evening person”) and “late chronotype” (including “evening person” and “more of an evening than a morning person”). Snoring information was collected using the question, “Does your partner, a close relative, or a friend complain about your snoring”. For this information, participants who answered “prefer not to answer” or “do not know” for any of the above questions were treated as having missing data.

A composite sleep risk score was calculated by summing the scores for all five sleep traits, yielding a total score ranging from 0 to 5, where a higher score indicated a poorer sleep pattern. Participants were categorized into three sleep pattern groups: “healthy sleep pattern” (0–1 poor sleep behaviors), “intermediate sleep pattern” (2–3 poor sleep behaviors), and “poor sleep pattern” (4–5 poor sleep behaviors).

### Measurement of NMR-based metabolomics

In the UK Biobank, blood samples were collected during the baseline assessment between 2007 and 2010 at 22 assessment centers across the UK^24^. Participants were not required to fast prior to sample collection; the mean interval since the last meal was approximately 4 hours^24^. The detailed protocols for blood sample collection and storage have been described elsewhere^24^. The metabolic biomarkers were measured between June 2019 and April 2020 using a high-throughput NMR metabolomics platform^25–27^. Detailed descriptions of the NMR platform and experimental procedures are available in the UKB documentation. Briefly, plasma samples were stored at -80 °C until processing, then gradually thawed at 4 °C overnight and centrifuged at 3,400 g for 3 min. Aliquots from each sample were transferred to NMR tubes and mixed with buffer before analysis. Metabolic profiling was conducted using a 500 MHz proton NMR spectrometer (Bruker AVANCE IIIHD), and biomarker quantification was performed using proprietary software developed by Nightingale Health.

The metabolomic analyses followed standardized quality control procedures jointly established by UK Biobank and Nightingale Health^27^. Each 96-well plate included two internal control samples and two blinded duplicates to ensure consistency^27^. The coefficients of variation for most biomarkers were below 5%, meeting the predefined quality standards^27^. Additionally, only approximately 1% of samples were affected by technical outliers, and their impact on epidemiological associations was considered minimal^27^. Finally, a total of 249 metabolic biomarkers were quantified per sample, comprising 168 direct measures and 81 percentage ratios. These biomarkers included routine lipids, fatty acids, inflammation markers, and various low-molecular-weight metabolites such as amino acids, ketone bodies, and glycolysis-related metabolites. For this study, metabolite ratio variables were excluded as they fell outside the scope of analysis, yielding a final set of 167 metabolites^28^. The details for these selected biomarkers are provided in Supplementary Table 1.

### Ascertainment of OA and its subtypes

OA cases were identified through record linkage to the National Health Service (NHS) central registries. The primary endpoint was defined based on individual medical records using International Classification of Diseases, 10th Revision (ICD-10) codes (Supplementary Table 2)^29^. Cases of inflammatory joint disease, infectious joint disease, and post-traumatic joint OA were excluded from this study^29^. The ICD-10 codes for hand OA, hip OA, and knee OA are provided in Supplementary Table 2. In this study, the follow-up time was determined based on the time from baseline to the first occurrence of OA diagnosis, death, loss to follow-up, or October 31, 2021, whichever occurred first.

### Statistical analyses

To improve the normality of the data, metabolite data were subjected to natural logarithmic transformation and subsequently standardized using Z-scores. The associations between sleep traits and OA were initially assessed using Spearman correlation coefficients. Cox proportional hazards regression models were then employed to estimate the effects of sleep traits on OA incidence. Model 1 was adjusted for age, sex (female and male) and race (white and other). Model 2 was additionally adjusted for Townsend deprivation index (TDI), education level (with university degree or not), and body mass index (BMI). Physical activity (yes or no), drinking status (never, previous and current), smoking status (never, previous and current), and sleep medicine used (yes or no) were further adjusted in the model 3. To support the snoring/apnea components of the sleep score, we examined the association between obstructive sleep apnea (OSA) (ICD-10 code: G47.3) and the risk of OA.

To investigate the metabolic profile associated with sleep patterns, we applied a LASSO regression model with ten-fold cross-validation, iterated ten times. In each iteration, participants were randomly divided into ten subsets, with one subset designated as the test set and the remaining nine as the training set. This process was repeated across all ten subsets^30^. No significant differences in sleep score, age, sex, BMI, or lifestyle factors were observed among these ten subsets (Supplementary Table 3). For the metabolites identified by LASSO regression across ten datasets, we further conducted a pathway enrichment analysis by MetaboAnalyst 6.0 (https://www.metaboanalyst.ca/)^31^. Pathways with a false discovery rate (FDR) corrected *P* value < 0.05 were considered statistically significant. A metabolomic score (Met-score) was derived as the weighted sum of metabolites selected by the LASSO model. Participants were subsequently categorized into quintiles based on their Met-score, and Cox proportional hazards regression models were used to evaluate the association between the sleep pattern–associated metabolomic profile and OA risk. Additionally, restricted cubic splines (RCS) with four knots were employed in the fully adjusted model to assess potential non-linear associations between the sleep score, Met-score, and OA risk.

**Fig. 1:**
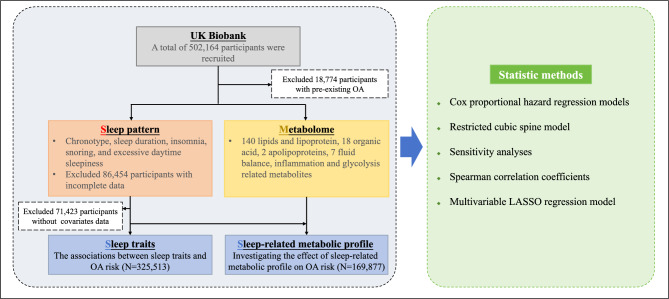
Overview of the study design and participant selection. A total of 325,513 participants were included in the analysis of associations between sleep traits and OA risk. Of these, 169,877 participants with available metabolic data were included in the subsequent analysis investigating the effect of sleep-related metabolic profile on OA risk. Abbreviation: OA, osteoarthritis.

Figure 1 presents an overview of the study design, illustrating the inclusion and exclusion criteria applied to the study population. After excluding individuals with pre-existing OA at baseline (N = 18,774) and those with incomplete data on sleep traits (N = 86,454) or covariates (N = 71,423), a total of 325,513 participants were included in the analysis. Of these, 169,877 participants with available metabolic data were included in the subsequent analysis investigating the effect of sleep-related metabolic profile on OA risk.

All analyses were performed by R version 4.2.1 and Python 3.12. Statistical significance was assessed using a Bonferroni correction for multiple comparisons, with a corrected threshold of *P* < 0.05/n, where n is the number of comparisons.

## Results

### Population characteristics

Over a mean follow-up period of 12.61 years, a total of 38,779 new OA cases were identified. Table 1 presents the baseline characteristics of participants, stratified by sleep patterns. Of the total cohort, 26,386 individuals were classified as having a healthy sleep pattern, while 63,252 participants were categorized as having a poor sleep pattern. Notably, those with a poor sleep pattern were male and had a higher likelihood of being current smokers. Additionally, these individuals were more likely to consume alcohol regularly and exhibited a tendency towards a higher BMI. In contrast, participants with a healthy sleep pattern were characterized by a lower BMI and were more likely to be non-smokers.

**Table 1:** The baseline information of included participants.

Characteristic	Health pattern, N = 26,386	Moderate pattern, N = 235,875	Poor pattern, N = 63,252
Age			
Mean (SD)	53.70 (8.38)	55.36 (8.04)	56.11 (7.90)
Sex			
Female	14,538 (55.10%)	135,512 (57.45%)	29,790 (47.10%)
Male	11,848 (44.90%)	100,363 (42.55%)	33,462 (52.90%)
White ethnicity			
No	1,429 (5.42%)	10,965 (4.65%)	4,057 (6.41%)
Yes	24,957 (94.58%)	224,910 (95.35%)	59,195 (93.59%)
University education			
No	14,491 (54.92%)	141,423 (59.96%)	39,727 (62.81%)
Yes	11,895 (45.08%)	94,452 (40.04%)	23,525 (37.19%)
BMI			
Mean (SD)	26.01 (4.16)	26.88 (4.55)	28.47 (5.09)
Smoking status			
Never	17,143 (64.97%)	136,132 (57.71%)	31,752 (50.20%)
Previous	7,425 (28.14%)	78,763 (33.39%)	23,392 (36.98%)
Current	1,818 (6.89%)	20,980 (8.89%)	8,108 (12.82%)
Drinking status			
Never	1,196 (4.53%)	8,401 (3.56%)	2,088 (3.30%)
Previous	864 (3.27%)	6,926 (2.94%)	1,939 (3.07%)
Current	24,326 (92.19%)	220,548 (93.50%)	59,225 (93.63%)
Physical activity			
No	8,460 (32.06%)	89,624 (38.00%)	27,436 (43.38%)
Yes	17,926 (67.94%)	146,251 (62.00%)	35,816 (56.62%)
TDI			
Mean (SD)	− 1.69 (2.87)	− 1.66 (2.89)	− 1.38 (3.06)
Sleep related medicine use			
No	26,111 (98.96%)	230,396 (97.68%)	61,273 (96.87%)
Yes	275 (1.04%)	5,479 (2.32%)	1,979 (3.13%)
OA incidence			
No	24,067 (91.21%)	208,234 (88.28%)	54,433 (86.06%)
Yes	2,319 (8.79%)	27,641 (11.72%)	8,819 (13.94%)

BMI, body mass index; OA, osteoarthritis; SD, standard deviation; TDI, Townsend deprivation index.

### Effect of sleep traits on OA risk

**Fig. 2:**
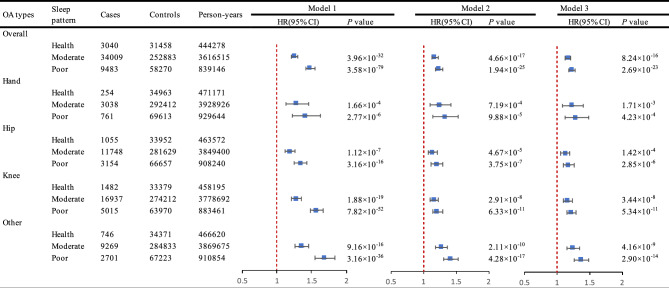
The association between sleep patterns and OA risk. Model 1 was adjusted for age, sex (female and male) and race (white and other). Model 2 was additionally adjusted for TDI, education level (with university degree or not), and BMI. Model 3 was additionally adjusted for physical activity (yes or no), drinking status (never, previous and current), smoking status (never, previous and current), and sleep medicine used (yes or no). Abbreviation: CI, confidence interval; HR, hazard ratio; OA, osteoarthritis.

Specifically, insomnia, shorter sleep duration, and frequent daytime sleepiness were all significantly associated with an increased likelihood of developing OA (*P* < 0.001), as detailed in Supplementary Table 4. This association remained across various OA subtypes, including hand OA, hip OA, and other types of OA. When further examining the association between OSA and OA risk, we found that participants with OSA at baseline had a higher risk of developing OA compared to those without OSA (Supplementary Table 5). When further explored the association between sleep patterns and OA, we found that individuals with poor sleep patterns showed a higher risk of OA (HR, 1.23, 95% CI, 1.18 to 1.28, *P* = 2.69 × 10^–23^). The risk of hand OA specifically was 1.29 times higher among those with poor sleep patterns compared to those with healthy sleep patterns (HR, 1.29, 95% CI, 1.12 to 1.49, *P* = 4.23 × 10^–4^). Similar relationships were observed for hip OA and other OA types, as presented in Fig. 2 and supplementary Table 6, where participants with poor sleep patterns consistently exhibited a higher risk compared to those with healthy sleep patterns. We also found that an increase in unhealth sleep score, associated positively with an elevated risk of OA and its subtypes (*P* for linearity < 0.001). There was no significant non-linearity association of sleep score with risk of OA and its subtype (Supplementary Fig. 1).

### Metabolic profile of sleep pattern and its effect on OA

**Fig. 3:**
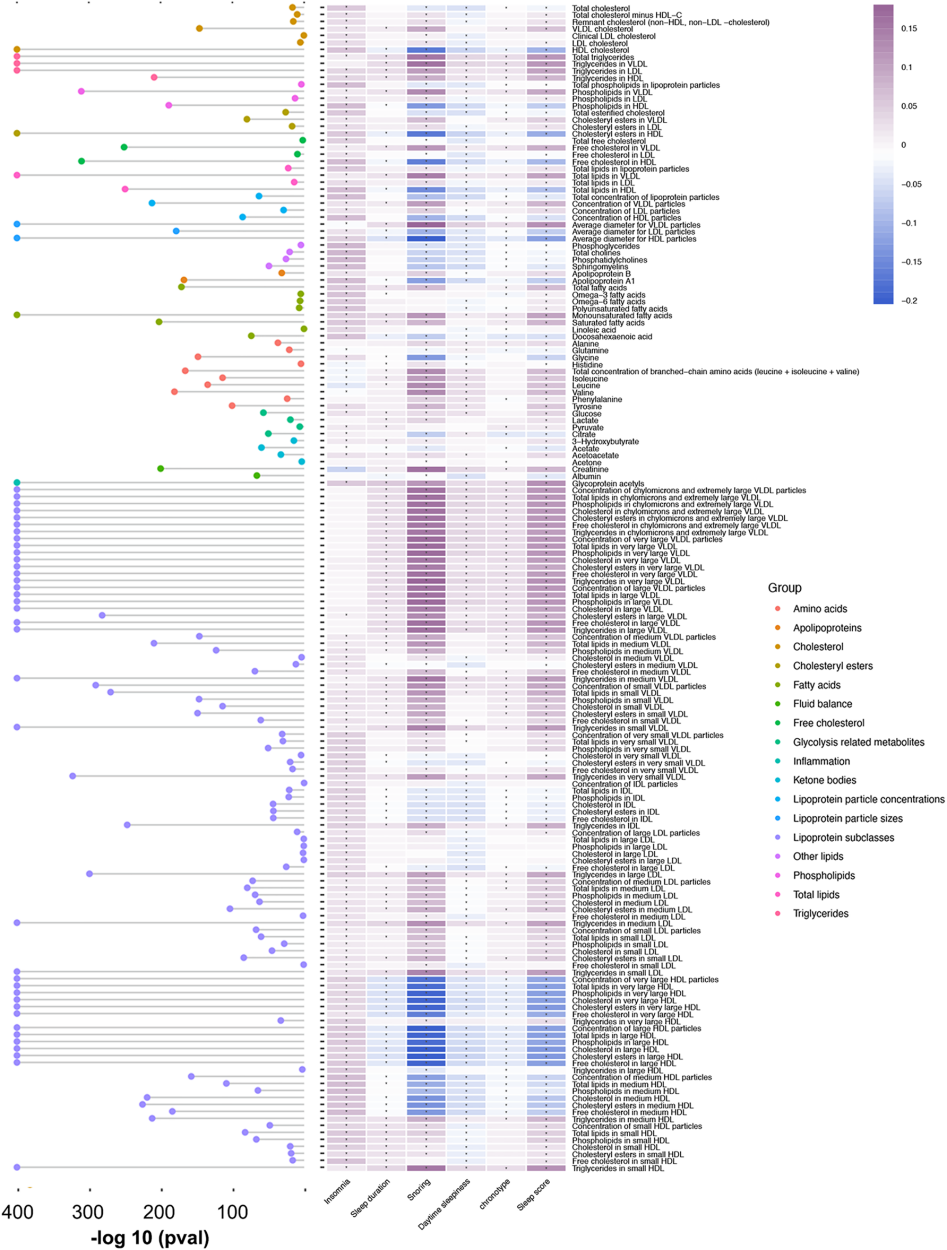
Correlation matrix and composition for sleep traits and all metabolic biomarkers. The Cleveland dot plot for associations between sleep score and all metabolic biomarkers is shown on the left side of the correlation matrix. The x-axis represents *P* value after logarithmic transformation, and the y-axis are these metabolites. * *P* < 0.05/167.

**Fig. 4:**
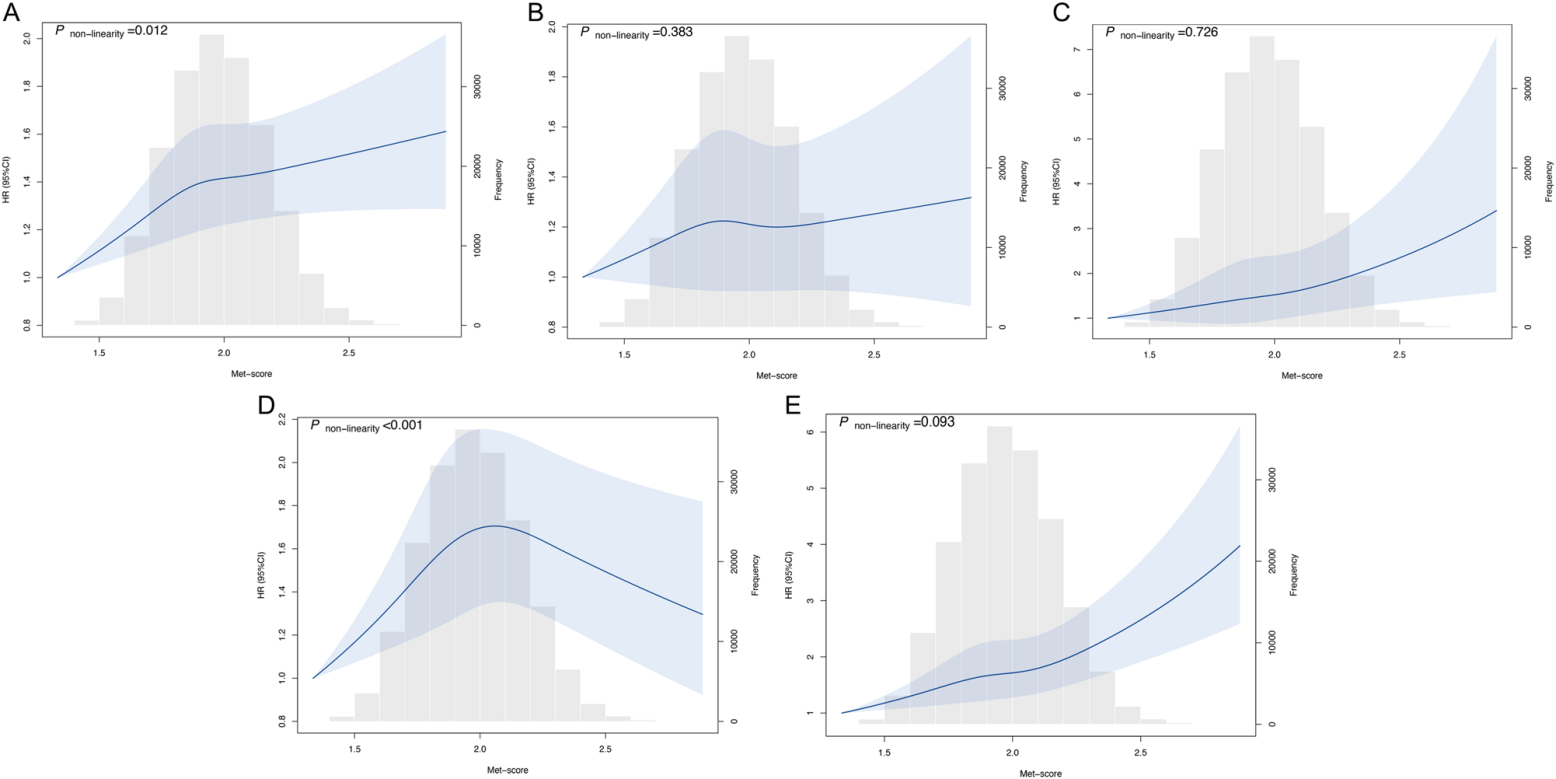
The dose–response relationship between metabolomic score and osteoarthritis risk. (**A**) Overall Osteoarthritis risk. (**B**) Hip osteoarthritis risk. (**C**) Hand osteoarthritis risk. (**D**) Knee osteoarthritis risk. (**E**) Other types of osteoarthritis risk. CI, confidence interval; HR, hazard ratio; Met-score, metabolomic score.

Our analysis of 167 metabolites found that 93% of these metabolites, a total of 155, displayed statistically significant correlations with baseline sleep scores (*P* < 0.05/167; Fig. 3). A total of 33 metabolites were selected by LASSO regression across ten datasets (Supplementary Table 7). Pathway enrichment analysis found that the most significantly enriched pathway was phenylalanine, tyrosine, and tryptophan biosynthesis (FDR-adjusted *P* = 0.031) (Supplementary Fig. 2). Upon examining the metabolic profiles associated with sleep patterns, we observed a consistent increase in the Met-score corresponding to higher sleep scores across all ten sets (Supplementary Fig. 3). Within the fully adjusted model, a positive and statistically significant association was observed between the Met-score and OA risk, with a *P*-value for trend of 1.10 × 10^–6^. Notably, individuals belonging to the highest quintile (Q5) of Met-score exhibited a 1.14-fold elevated risk of OA compared to those in the lowest quintile (HR, 1.14; 95% CI, 1.08 to 1.20; *P* = 3.09 × 10^–6^). The similar risk effect of Met-score on hand and knee OA risk was found (Supplementary Table 8). Subsequent application of RCS regression analysis revealed a non-linear dose–response relationship between the Met-score and knee OA risk (*P* < 0.001, Fig. 4).

## Discussion

In this prospective study, we integrated five sleep traits-daytime sleepiness, insomnia, sleep duration, chronotype, and snoring-to develop a comprehensive sleep pattern. We analyzed the associations between each individual sleep trait, as well as the overall sleep pattern, and OA using data from a large cohort. Our findings revealed that participants with a poor sleep pattern had a 23% higher risk of OA compared to those with a healthy sleep pattern. Additionally, we identified a metabolomic signature linked to our sleep score and examined its association with OA risk. Participants classified with a healthy metabolomic signature had a lower risk of developing OA than those with an unhealthy metabolic status in the fully adjusted model. Unlike most previous studies, our study conducted prospective analyses and accounted for the combined effects of various sleep behaviors on OA risk. For the first time, we employed a metabolomic approach to objectively characterize a healthy sleep pattern, providing new insights into the potential mechanisms linking sleep with OA risk.

In recent years, several observational studies have investigated the associations between sleep behaviors and the risk of OA. For instance, a nationwide cross-sectional observational study found that both short (≤ 5 h/day) and long (≥ 9 h/day) sleep duration was positively associated with osteoarthritis in middle-aged and older women^32^. Another recent study found that poor nighttime sleep quality, especially participants with 5–7 sleep restless days/week were associated with significantly increased risk of incident knee OA among middle-aged and older adults in China^33^. Furtherly, using univariable and multivariable Mendelian randomization (MR) methods, Ni et al.^8^ found genetic evidence that both insomnia and short sleep duration were associated with OA risk. Nonetheless, the majority of earlier research evaluated the individual influence of one single sleep behavior on OA risk, ignoring the combined effects of multiple sleep behaviors in real-world settings. Our study created a sleep risk score and further support the association between sleep traits with OA risk which consistent with previous findings. These results further emphasize that maintaining a favorable sleep pattern can significantly contribute to the primary prevention of OA.

While the exact mechanism underlying the observed association between sleep pattern and OA is not fully understood, possible explanation can be suggested through metabolism. In our study, we found that sleep patterns may influence the biosynthesis of phenylalanine, tyrosine, and tryptophan, which have previously been implicated in the development of OA^34,35^. Sleep traits may lead to metabolic dysregulation, which could contribute to an increased risk of OA^28^. In brief, phenylalanine and tyrosine are precursors for catecholamines and pro-inflammatory mediators, and altered levels may exacerbate low-grade inflammation and cartilage degradation in OA^36,37^. Sleep disturbances can influence mental and physiological states, altering the metabolism and consequently leading to the occurrence of autoimmune diseases^38^. Additionally, recent MR studies have revealed that insufficient sleep increased the risk of obesity, which was also positively associated with OA risk^39^. Adipose tissue releases fatty factors that elevate pro-inflammatory mediators, serving as a mechanism through which obesity exerts damaging effects on joint tissues^40^. Based on this assumption, we regressed sleep score on the 167 metabolomic biomarkers using LASSO regression, aiming to develop a metabolomic signature that objectively captures the changes in the plasma metabolites associated with overall sleep patterns. This approach may avoid the recall bias effect of self-reported information and offered an objective and insight method to examining the relationship between sleep behaviors and OA risk.

Our study has some strengths. First, it was a large prospective cohort study based on data from the UK Biobank. Second, the availability of various covariates allowed us to adjust for many potential confounders related to OA and sleep patterns. In addition, the metabolomic signature provided an objective method for defining sleep patterns and offered new insights into the mechanisms linking sleep to OA. However, this study also has some limitations. First, the assessments of sleep behaviors were based on self-reports from participants, which could affect the accuracy of the results. To address this issue, we conducted a sensitivity analysis including ICD-coded OSA and observed a similar association with OA risk. Due to the limited number of cases and potential underreporting of OSA in the UKB, this analysis should be considered exploratory and requires further validation. Second, some unmeasured confounding factors may potentially affect the results. Third, although this study employed a prospective design and excluded participants with OA at baseline, the possibility of reverse causality cannot be fully ruled out, as OA-related symptoms may contribute to sleep disturbances. Therefore, the causal relationship between sleep patterns and OA risk warrants further investigation. In addition, as sleep information was collected only at baseline, potential time-varying changes in individual sleep traits were not accounted for in the analysis. This limitation may have resulted in exposure misclassification, potentially biasing the observed associations and underestimating the true effect sizes. Furthermore, this analysis focused on metabolites measured from samples collected at baseline, preventing the evaluation of time-varying associations with risk of OA. Finally, this study was based on the UK Biobank, which predominantly includes white participants, potentially limiting the generalizability of the results to other populations.

In conclusion, our findings offer important public health insights by incorporating a multidimensional evaluation of self-reported sleep patterns and identifying novel associations between sleep behaviors and OA. Although our study does not employ objective or clinically validated sleep assessments, the use of multiple sleep-related dimensions enhances the comprehensiveness of exposure characterization. These results provide a basis for further investigation into the behavioral and biological mechanisms linking sleep and OA risk.

## Supplementary Information

Supplementary Information 1.

Supplementary Information 2.

Supplementary Information 3.

Supplementary Information 4.

Supplementary Information 4.

## Data Availability

The datasets generated during and/or analysed during the current study are available in the UK Biobank, www.ukbiobank.ac.uk.
