# Supplementary figures and images for: Sleep patterns, plasma metabolites, and risk of incident osteoarthritis: a prospective cohort study
Source: Sci Rep. 2025 Aug 11;15:29334. doi: 10.1038/s41598-025-07711-1 (PMC12339921; doi:10.1038/s41598-025-07711-1)

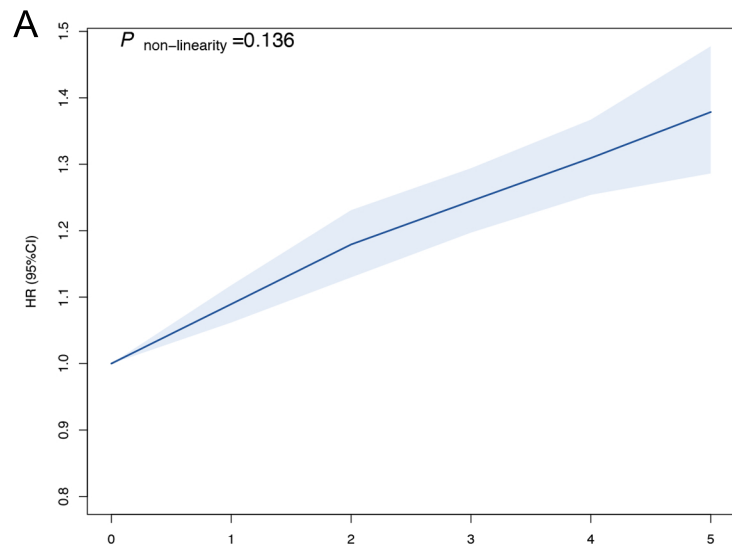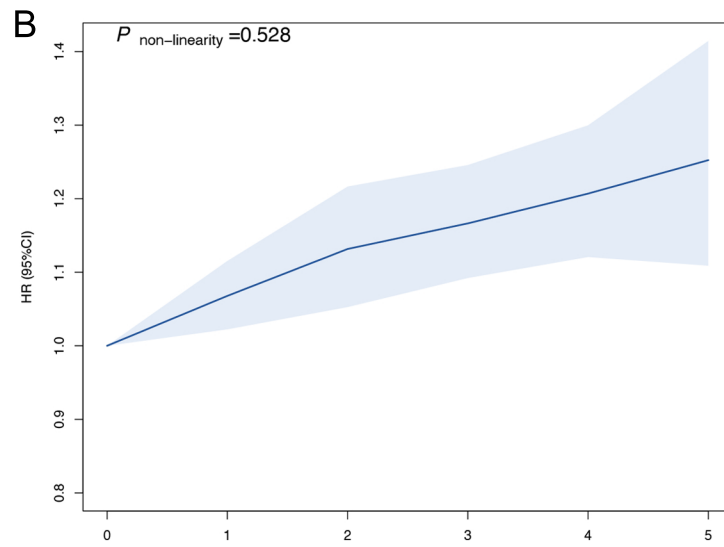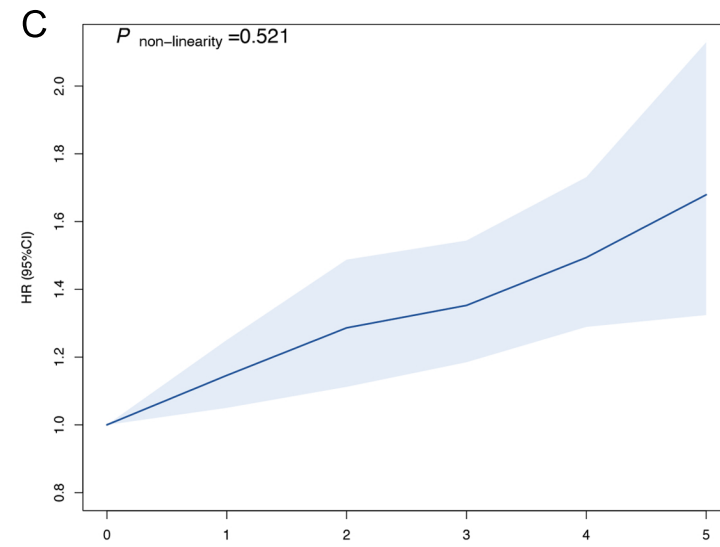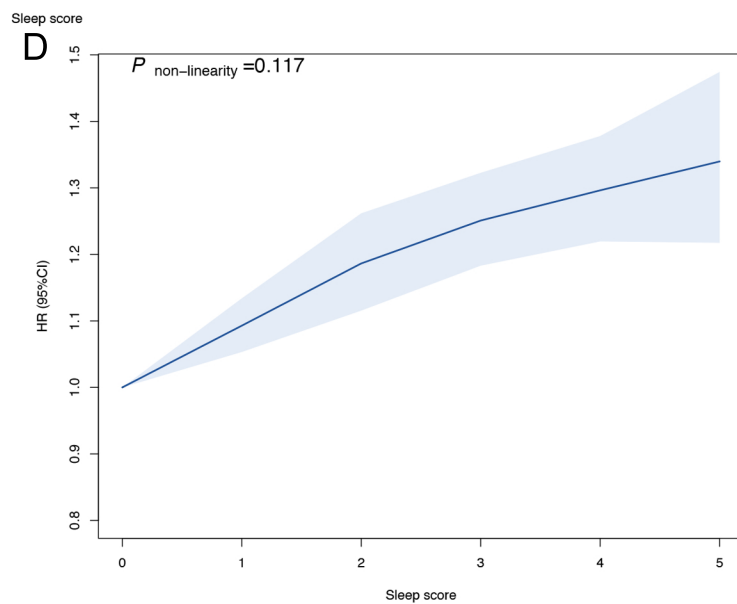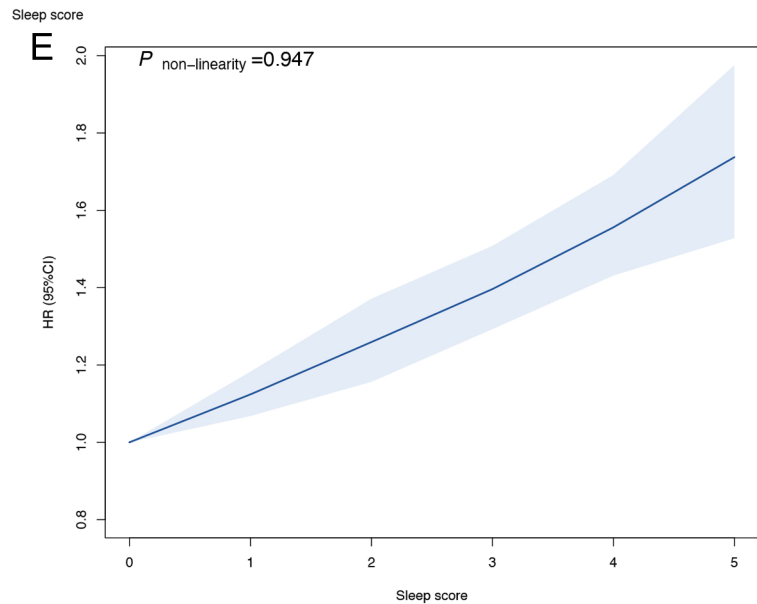

Supplement: Supplementary file 1 — Supplementary Information 1. [file 41598_2025_7711_MOESM1_ESM.pdf]

# Overview of Enriched Metabolite Sets (Top 25)

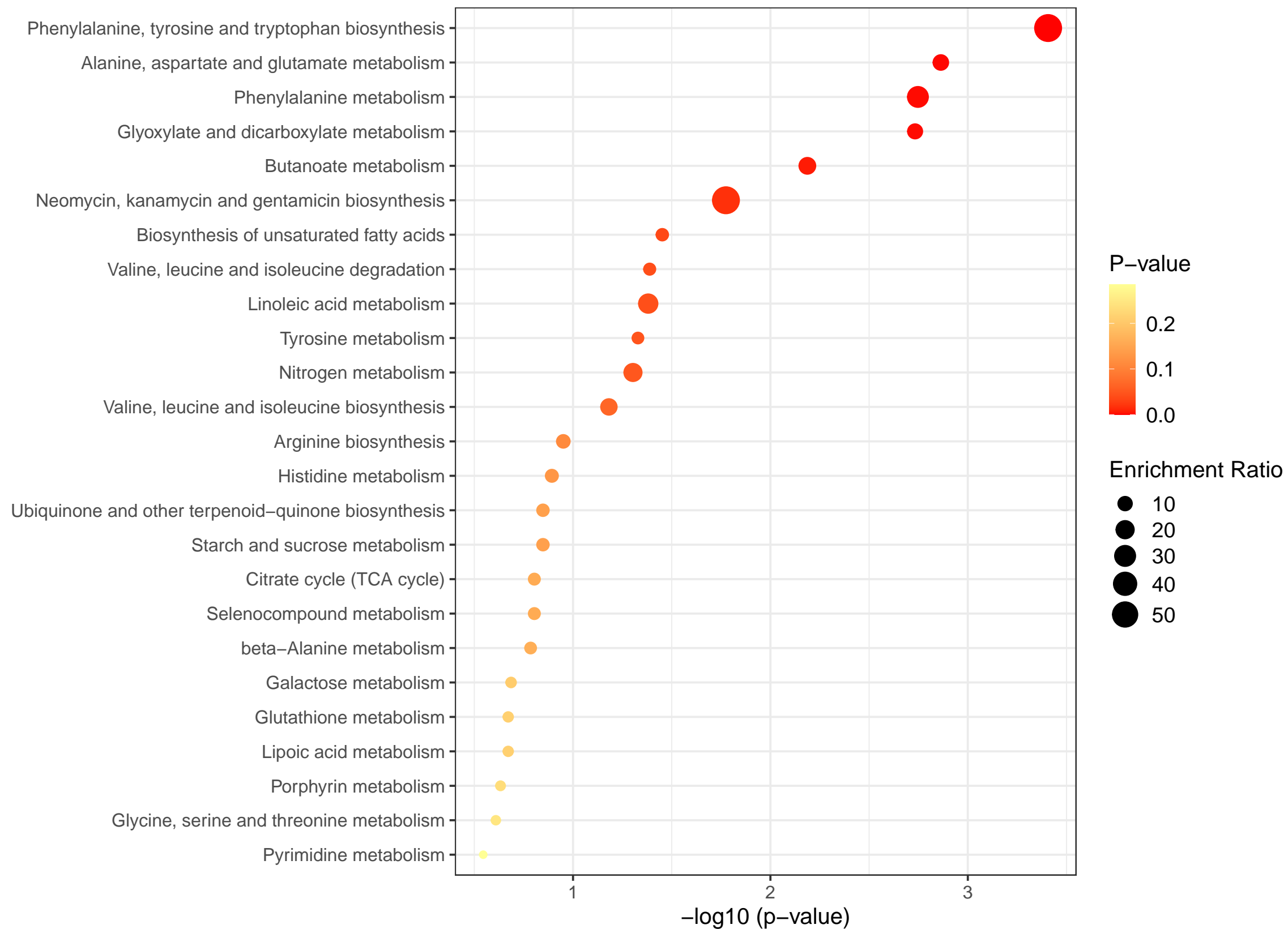

Supplement: Supplementary file 2 — Supplementary Information 2. [file 41598_2025_7711_MOESM2_ESM.pdf]

R-squared: 0.406

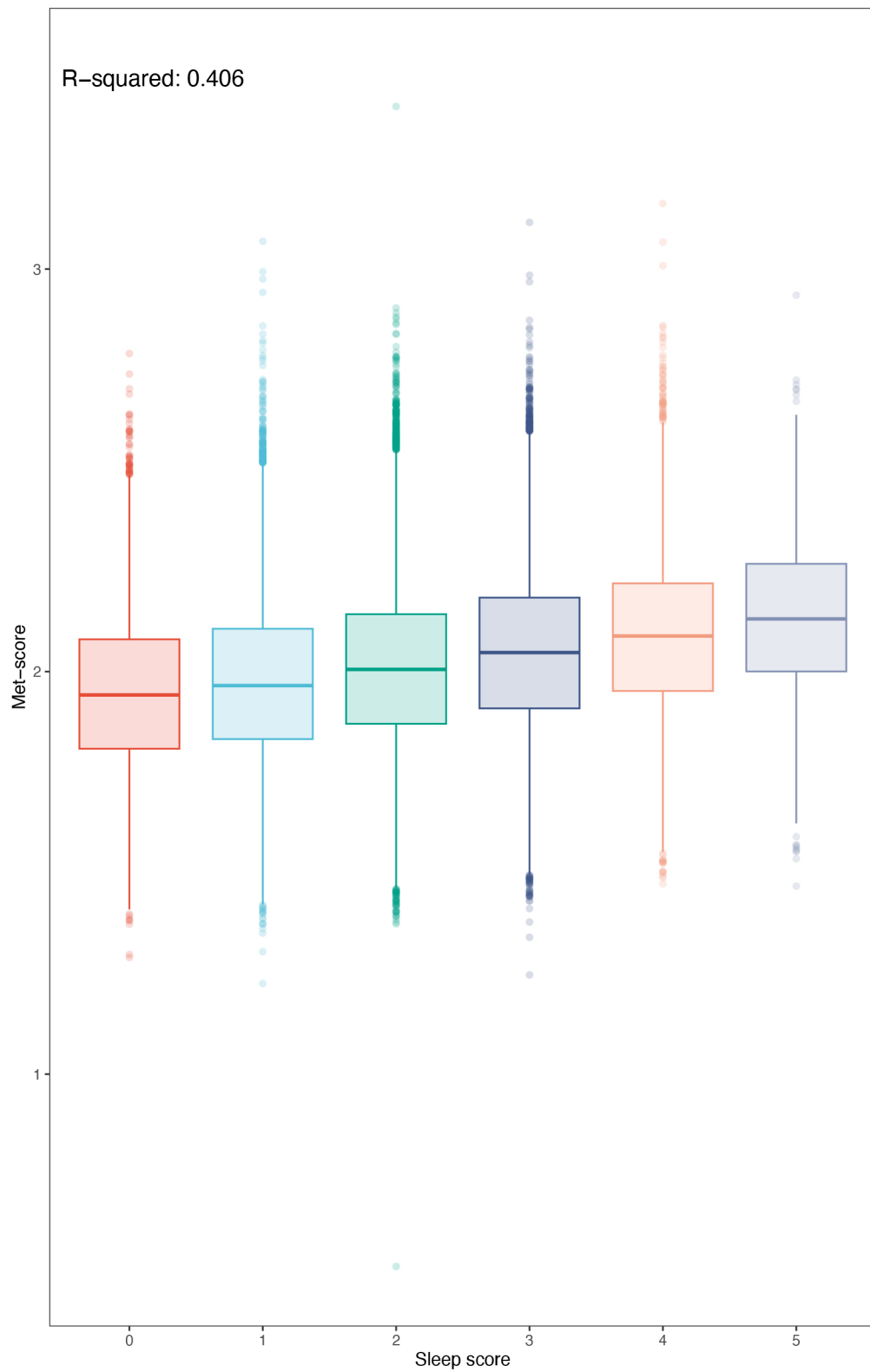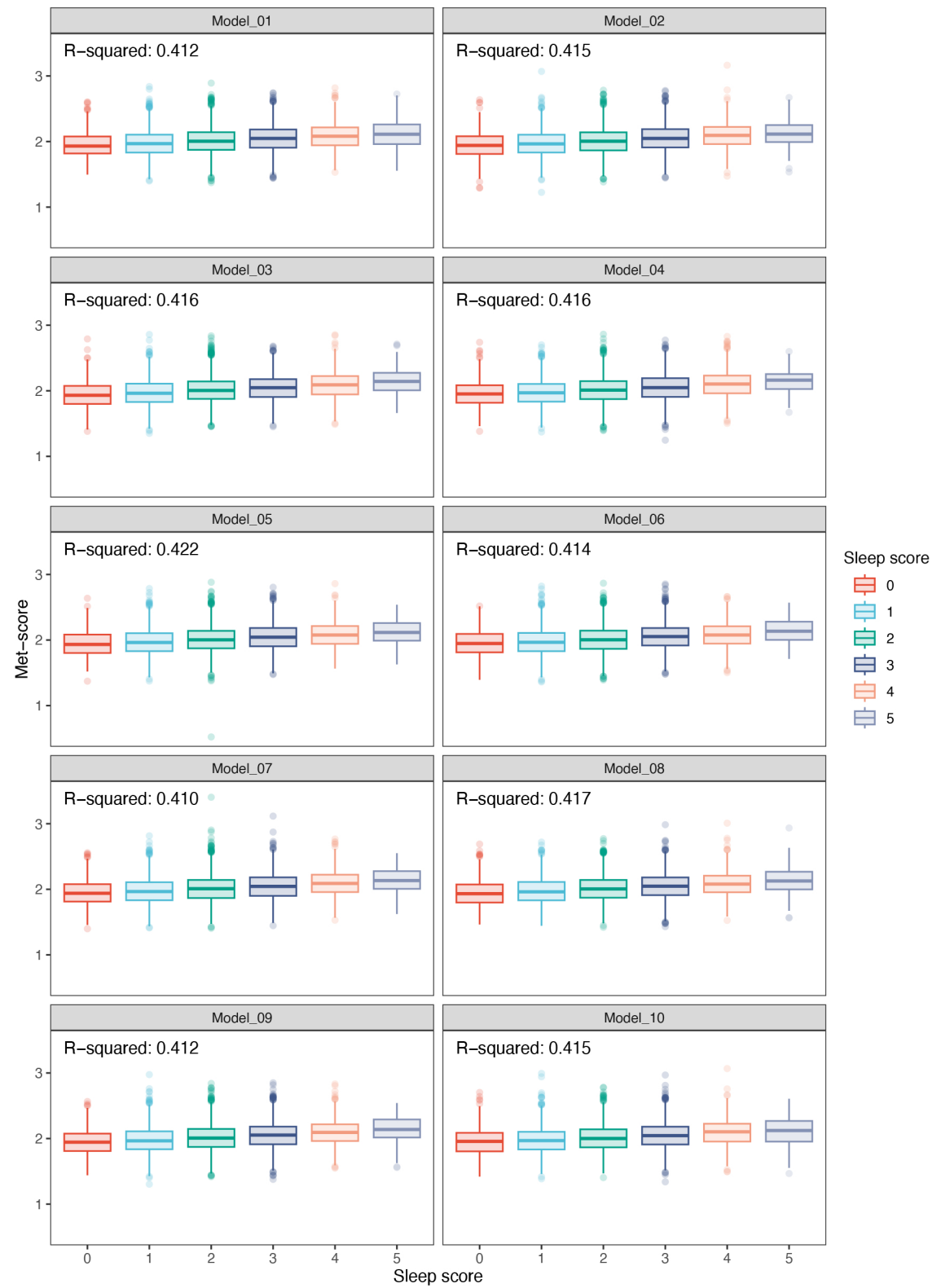

Supplement: Supplementary file 3 — Supplementary Information 3. [file 41598_2025_7711_MOESM3_ESM.pdf]
