# Supplementary material for: Sleep patterns, plasma metabolites, and risk of incident osteoarthritis: a prospective cohort study
Source: Sci Rep. 2025 Aug 11;15:29334. doi: 10.1038/s41598-025-07711-1 (PMC12339921; doi:10.1038/s41598-025-07711-1)
Supplement: Supplementary file 5 — Supplementary Information 4. [file 41598_2025_7711_MOESM5_ESM.docx]

**Supplementary figure legends**

**Supplementary Figure 1. The dose-response relationship between sleep score and osteoarthritis risk.** (A) Overall Osteoarthritis risk. (B) Hip osteoarthritis risk. (C) Hand osteoarthritis risk. (D) Knee osteoarthritis risk. (E) Other types of osteoarthritis risk.

Abbreviation: CI, confidence interval; HR, hazard ratio; Met-score, metabolomic score.

**Supplementary Figure 2. Pathway enrichment analysis was conducted based on 33 metabolites identified by LASSO regression across ten datasets.** The x-axis represents *P* value after logarithmic transformation, and the y-axis are pathways.

**Supplementary Figure 3. The correlation between sleep score and metabolomic score.** The x-axis represents the sleep score, calculated based on multiple sleep traits, while the y-axis indicates the metabolomic score derived from LASSO regression
